# The Fukushima Health Management Survey: estimation of external doses to residents in Fukushima Prefecture

**DOI:** 10.1038/srep12712

**Published:** 2015-08-04

**Authors:** Tetsuo Ishikawa, Seiji Yasumura, Kotaro Ozasa, Gen Kobashi, Hiroshi Yasuda, Makoto Miyazaki, Keiichi Akahane, Shunsuke Yonai, Akira Ohtsuru, Akira Sakai, Ritsu Sakata, Kenji Kamiya, Masafumi Abe

**Affiliations:** 1 Radiation Medical Science Center for the Fukushima Health Management Survey, Fukushima Medical University; 2 Department of Epidemiology, Radiation Effects Research Foundation. 5-2 Hijiyama Park, Minami-ku, Hiroshima City, 732-0815 Japan.; 3 National Institute of Radiological Sciences. 4-9-1, Anagawa, Inage-ku, Chiba City, 263-8555 Japan.; 4 Research Institute for Radiation Biology and Medicine, Hiroshima University.

## Abstract

The Fukushima Health Management Survey (including the Basic Survey for external dose estimation and four detailed surveys) was launched after the Fukushima Dai-ichi Nuclear Power Plant accident. The Basic Survey consists of a questionnaire that asks Fukushima Prefecture residents about their behavior in the first four months after the accident; and responses to the questionnaire have been returned from many residents. The individual external doses are estimated by using digitized behavior data and a computer program that included daily gamma ray dose rate maps drawn after the accident. The individual external doses of 421,394 residents for the first four months (excluding radiation workers) had a distribution as follows: 62.0%, <1 mSv; 94.0%, <2 mSv; 99.4%, <3 mSv. The arithmetic mean and maximum for the individual external doses were 0.8 and 25 mSv, respectively. While most dose estimation studies were based on typical scenarios of evacuation and time spent inside/outside, the Basic Survey estimated doses considering individually different personal behaviors. Thus, doses for some individuals who did not follow typical scenarios could be revealed. Even considering such extreme cases, the estimated external doses were generally low and no discernible increased incidence of radiation-related health effects is expected.

Following the Fukushima Dai-ichi Nuclear Power Plant (Fukushima NPP) accident, the Fukushima Prefectural Government and Fukushima Medical University started a health management survey for all Fukushima residents (about two million people) in 2011^1^,^2^. It is called the Fukushima Health Management Survey and consists of a “Basic Survey” and four detailed surveys, namely the Thyroid Ultrasound Examination, Comprehensive Health Check , Mental Health and Lifestyle Survey, and Pregnancy and Birth Survey. For the Health Management Survey, external dose values are considered to be necessary data for long-term health management. There are three points to be considered for estimating external dose in the initial stage after the accident: (1) gamma ray dose rates varied considerably from place to place in Fukushima Prefecture; (2) most of the people who were living in the Evacuation Area and the Deliberate Evacuation Area moved from their original locations after the accident; and (3) such moves were different from person to person. Thus, records of the movements and activities on an individual level need to be taken into consideration when estimating the “initial dose” after the accident.

Since the Fukushima NPP accident, many dose estimation studies have been done and two reviews are available^3^,^4^. In particular, the municipalities of Fukushima Prefecture have widely conducted external dose estimations by using passive dosimeters distributed to residents upon their request^5^. However, such activities were generally started several months after the accident. Most of the studies on estimating the initial dose after the accident made assumptions on the whereabouts, including preparing daily time-budgets (time spent indoors or outdoors), of residents^6^,^7^,^8^,^9^.

For example, a report by the WHO states that in the two most affected locations in Fukushima Prefecture, Namie and Iitate, the preliminary estimated radiation effective doses for the first year ranged from 12 to 25 mSv (including internal exposure)^9^. The estimated doses were based on “conservative” assumptions. An example of one of these assumptions was that relocation in the Deliberate Evacuation Area took place at 4 months, although the inhabitants of this area were subjected to relocation at different times previous to this.

The UNSCEAR 2013 report, published in April 2014, well summarizes the doses due to the accident^10^. The UNSCEAR Committee applied typical evacuation scenarios for estimating external doses for evacuees^11^. As for non-evacuees, they used measurement datasets of the deposition density of radionuclides on the ground, and some computational models^4^. Since personal behaviors such as whereabouts and daily time-budgets were not considered for the dose estimation, only district or settlement average doses were given in the UNSCEAR report.

The Basic Survey is characterized by estimating external doses on a personal basis^1^. For this purpose, an individual dose estimation system was developed by the National Institute of Radiological Sciences (NIRS)^11^. The individual doses are estimated using the system and the behavior data collected from the residents by Fukushima Medical University. This paper describes distributions of the individual doses, including distributions by regions, age groups and sex.

## Methods

### Questionnaire for the Basic Survey

The study protocol of the Basic Survey was reviewed and approved by the Ethics Review Committee of Fukushima Medical University. The study was conducted in accordance with the approved guidelines. By sending back the response sheet, the respondent is deemed to have read and agreed the purpose statement for the questionnaire of the Basic Survey. In the case of children, consent is deemed to be obtained by their guardians’ signature on the response sheet.

**Figure 1:**
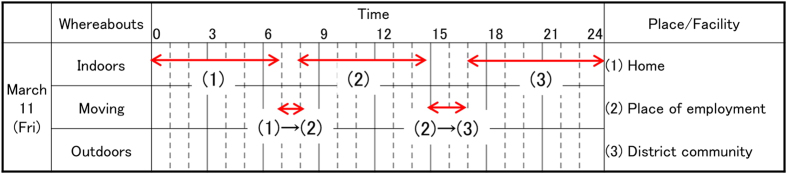
An example form for writing records of moves and activities in the Basic Survey questionnaire.

**Figure 2:**
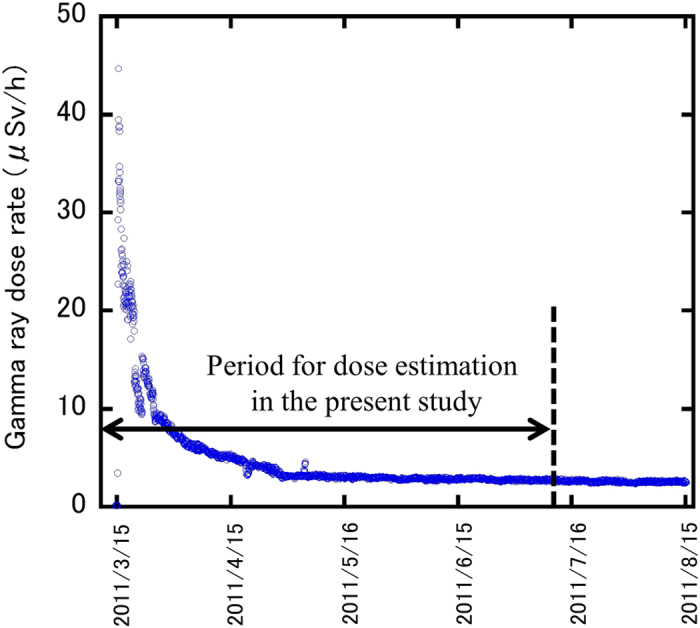
Changes in gamma ray dose rate at a monitoring station near the Iitate Village hall.

The self-administered questionnaire was prepared in order to collect information from residents on their dwelling place, places visited, length of time spent indoors and outdoors, and travelling time during the period from March 11 to July 11. The respondents were asked to fill in the questionnaire form for the period from March 11 to March 25; an example of a completed form for one day’s activities is given in Fig. 1. A complete form of the questionnaire is shown in Supplementary information (Sheet S1). The period from March 11 to March 25 was when the atmospheric radiation dose was the highest (Fig. 2). For the later period to July 11, a simpler form was used. As shown in Fig. 1, three columns were prepared corresponding to categories of activities: being inside of a building (shielding is considered depending on the types of building), being outside, and moves. In the case of moves, shielding by the vehicle was not considered in the system^11^. After filling out the form, respondents were asked to mail it back.

The original target population of the Basic Survey was 2,055,533, based on the number of registered residents in Fukushima Prefecture during the period of March 11 to July 1 (including those evacuated or who transferred their residence registration to another prefecture). Sending questionnaires to the original target population began in June 2011 and ended in October 2011.

As an additional service, the questionnaires were sent to persons falling under any of the following categories at their request; (1) a resident of Fukushima Prefecture who was registered in another prefecture during the period of March 11 to July 1; (2) a resident of another prefecture who commuted to Fukushima Prefecture during the period of March 11 to July 1; and (3) a resident of another prefecture who temporarily stayed in Fukushima Prefecture during the period of March 11 to 25. The number of additional-service questionnaires sent was 3,823 as of June 30, 2014. The responses from these persons were processed for individual dose estimation (details are described later) as a health care service, but excluded from further analysis shown in Tables 1, 2, 3, 4.

For children of elementary school age or younger, their parents were asked to fill in the form instead. Additionally, for children under the age of 20, parents were asked to sign the questionnaire and verify the information. The questionnaire included an item to ask if the respondent had been a radiation worker or not. Although individual doses were estimated for radiation workers (including those formerly employed as radiation workers), they were excluded from further analysis (Tables 1, 2, 3, 4).

To increase the response rate, a simplified questionnaire was prepared in November, 2013. The simplified questionnaire does not require respondents to fill in their detailed behavior in the form shown in Fig. 1. However, the target of the simplified questionnaire is residents who have not relocated or who have made only a single relocation. The number of responses (response rate) mentioned hereafter includes the number of responses to the simplified version of the questionnaire. As a result of cross-checking between the individual doses estimated with both questionnaires for 91 volunteers, no significant difference was seen. Thus, the individual doses estimated with the simplified version were compiled together in Tables 1, 2, 3, 4.

### Checking of collected responses to the questionnaire

The number of total responses obtained as of June 30, 2014 was 541,653 from the original target population (the number of responses from the questionnaires sent as the additional service was not included). Efforts to collect the responses continue to be made and responses were still being mailed back even at the time of writing in 2015. Of the total responses (541,653), individual dose estimation has been completed for 508,388 responses, as of June 30, 2014. There are two main reasons for the difference in number between the total responses and the responses with completed dose estimation. Firstly, since the collection of the responses is still ongoing, the recent collected responses were counted in the number of total responses, but were being processed in steps before dose estimation (digitization of response, etc).

Secondly, some of the responses had lack of information for estimating individual dose. The responses obtained were checked as to whether the contents were good enough for dose calculation. For example, if the name of the place where a respondent stayed on a given day was not clearly given on his/her response sheet, the location of the place could not be converted to longitude and latitude, which means dose estimation was not possible (see the next section).

In the case of incomplete responses, staff members of Fukushima Medical University contacted the respondents by telephone or mail to obtain the necessary information for dose calculation. The number of such cases amounted to more than 70,000. Still, in some cases, respondents could not be contacted as of June 30, 2014, mainly due to incomplete contact information. Such responses were not brought into the process of individual dose estimation, but included in the number of responses. Among the responses (508,388) for which individual dose estimation was possible, there were some for which periods with records of behavior data were less than four months for certain reasons. For most of such cases, the columns corresponding to March 11 through some point before July 11 were filled in. Although individual doses were also calculated for those cases, they were excluded from further analysis (Tables 1, 2, 3, 4 and S1, details are shown in Result section).

### Digitization of questionnaire and dose estimation

The responses to the questionnaire were filled out by hand. Trained staff performed data entry from the responses into electronic files. The information on addresses or names of locations (residence places, workplaces, schools, etc.) was converted to latitude and longitude. Hour-by-hour changes in the latitude and longitude were estimated from the relocation records. Types of buildings and the floor where a person stayed were also converted to codes. The electronic files including such kind of information were then converted to the digitized form readable by a computer program developed by NIRS.

Then, individual doses were estimated using the NIRS computer program^11^. The program was designed to calculate individual effective doses by superimposing the behavior data of the residents with daily gamma ray dose rate maps. A brief description of the program is given below:

In the program, it was assumed that the individual doses were received from: (1) airborne radioactive materials emitted by the accident for a period from March 12 to 14; and (2) the radioactive materials deposited on the ground for the period of March 15 and after, due to rain or snowfall on March 15 in Fukushima. Although ISO (isotropic irradiation) geometry was adopted for both periods, different shielding factors for each type of building were set for each. The shielding factors for “cloud source geometry” were set for the period of March 12 to 14 and those for “surface deposition geometry” were set for the period of March 15 and after. As an example, the shielding factor for a one or two story wooden-frame house was 0.9 for the “cloud source geometry”, while it was 0.4 for the “surface deposition geometry”. The individual doses were calculated for three different activities: staying indoors (shielding factors were set by different types of buildings), staying outdoors, and moving from one place to another. Doses during moves were calculated by multiplying time spent for the move and mean effective dose rate of start points and end points. No shielding was assumed during moves.

The program has daily (each day from March 12 to July 11, 2011) gamma ray dose rate maps for all parts of Fukushima Prefecture and a part of the neighboring four prefectures. Some evacuees left Fukushima Prefecture during the four months of dose estimation. In such cases, doses during the stay in other prefectures (including the neighboring four prefectures) were set at zero. If an evacuee returned to Fukushima Prefecture before July 11, the days up to July 11 were added to the doses calculated during the stay in Fukushima Prefecture before the evacuation. The release of radioactive materials from Fukushima Daiichi Nuclear Power Plant began on March 12, 2011, the day after the Great East Japan Earthquake. The dose on March 11 was therefore considered as zero, although the respondents were asked to fill in their behaviors from March 11. This is because the behavior records from 0 a.m. of March 12 (12 p.m. of March 11) was necessary for the dose calculation.

The daily dose rate maps were constructed based on two types of data. One is the hourly effective dose rate maps simulated by SPEEDI with the source term calculated using the MELCOR code by the Nuclear and Industrial Safety Agency (NISA), which was performed between March 12 and 14, 2011. The number of measurement points was not sufficient to construct the dose rate maps during that period. At present, we have no alternatives for first three days because of the lack of monitoring data.

Another data source is the monitoring data released by the Ministry of Education, Culture, Sports, Science and Technology (MEXT), which was used between March 15 and July 11, 2011. Since the government officially reported these data, they were considered to be most reliable among the available data.

In the program, dose due to natural background radiation was not included in the estimated dose. The contributions of natural background radiation were estimated in the following way: The Fukushima Prefectural government reported the averaged air kerma rates per month of 23 points in the prefecture to be 33–54 nGy/h before the accident. The other measured data showed 0.04 μGy/h in Fukushima City (representative of the Kempoku area), 0.04–0.06 μGy/h in Koriyama City (representative of the Kenchu area), 0.04–0.05 μGy/h in Shirakawa City (representative of the Kennan area), 0.04–0.05 μGy/h in Aizuwakamatsu City (representative of the Aizu area), 0.02–0.04 μGy/h in Minami-Aizu Town (representative of the Minami-Aizu area), 0.05 μGy/h in Minami-soma City (representative of the Soso area) and 0.05–0.06 μGy/h in Iwaki City (representative of the Iwaki area). Since the median value among them was 0.04 μGy/h (0.03 μSv/h as effective dose rate), it was used for subtracting contribution of natural background radiation dose rate. The background dose rate of 0.03 μSv/h corresponds to a four-month effective dose of 0.05 mSv, assuming a shielding factor of 0.4 for wooden houses and a daily time-budget (outdoors: 8 h and indoors: 16 h)^12^. Since it is considered to be within a range of uncertainty (details are described in the Discussion section), the variation of background radiation has little effect on the estimation of individual dose.

Dose conversion coefficients from ambient dose equivalent to effective dose are different between children and adults due to body size. Thus, the conversion coefficient was corrected as a function of age for children (≦15 y) in the present dose calculation. The estimated individual dose has been mailed to each respondent by Fukushima Medical University. In addition, the results of the Basic Survey have been updated and reported periodically at meetings held by the Fukushima Prefectural Oversight Committee^13^.

## Results

### Characteristics of responses to the questionnaire

**Figure 3:**
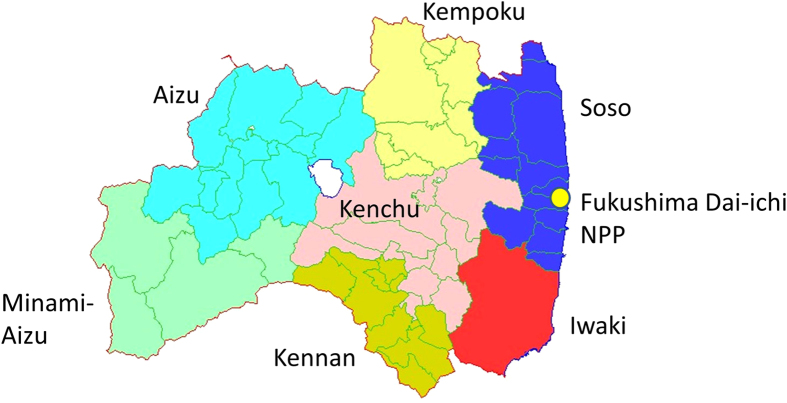
Map showing the location of the Fukushima Dai-ichi NPP in relation to seven areas of Fukushima Prefecture. The map was created by Haku-chizu KenMap software (http://www5b.biglobe.ne.jp/t-kamada/CBuilder/kenmap.htm, freeware approved by Geospatial Information Authority of Japan with approval No. 149 in 2002)^14^. The white unnamed area in the center is Lake Inawashiro.

**Figure 4:**
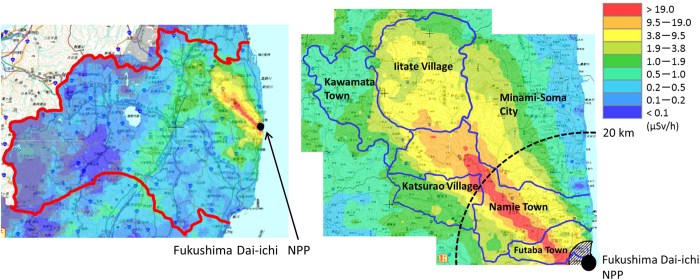
Gamma ray dose rate maps of Fukushima Prefecture These maps were modified by using PowerPoint® and Adobe Reader® software, from maps obtained by airborne monitoring surveys made in April to June, 2012^15^. No map available on the “Extension Site of Distribution Map of Radiation Dose, etc.,/Digital Japan” (http://ramap.jmc.or.jp/map/eng/) shall be quoted in any other documents without explicitly referring to the site as the source of the map. Decay correction was made for June 28, 2012. (**a**) The whole prefecture map and (**b**) a blown-up map focusing on the highest dose rate areas.

The overall response rate to the original population for the Basic Survey was 26.4% (541,653/2,055,533) as of June 30, 2014. Figure 3 is a map showing the location of the Fukushima Dai-ichi NPP in relation to the seven areas^14^. Regional variations in the response rates were observed: 19.6% (Minami-Aizu); 20.0% (Aizu); 21.2% (Kennan); 23.4% (Kenchu); 24.1% (Iwaki); 29.1% (Kempoku); and 45.3% (Soso). In the Soso area, Namie Town had the highest response rate (60.3%). The difference in response rate between the areas may also reflect the concern of the participants based on their understanding of possible exposure to a higher gamma ray dose rate level, as shown in Fig. 4(a)^15^. The Minami-Aizu area had the lowest response rate (19.6%), which corresponds to the lowest gamma ray dose rate (Figs 3 and 4(a)). The highest response rate was seen in the Soso area, which had the highest gamma ray dose rate level.

The dose distribution by area (Table 1) is already accessible online to the public^13^. The residents already know the level of the four-month individual doses in their residential areas. This may possibly be the reason for the submission of responses not being well promoted, especially in the low dose areas. Thus, some residents may not feel that knowing their individual doses by submitting the responses is necessary.

### Individual dose estimation

Doses have been estimated for 508,388/541,653 respondents (93.9%) as of June 30, 2014. Table 1 shows the dose distribution by area for a total of 421,394 respondents. Here, a total of 8,682 radiation workers and 78,312 respondents with behavior data for less than four months were excluded from the number of completed dose estimations (508,388). Individual doses for 421,394 persons shown in Table 1 are all four-month doses (March 11 to July 11, 2011), including the average and maximum doses. The distribution of respondents for which dose estimation was carried out (excluding radiation workers) by age group is shown in Table 3. The number of responses with completed dose estimates for each age group was larger for children than adults, as seen in this table.

The doses for more than 88% of the respondents in the Kempoku area and more than 93% of the respondents in Kenchu area were less than 2 mSv. The doses for approximately 89% of the respondents in the Kennan area and more than 99% of those in the Aizu and Minami-Aizu areas were less than 1 mSv. Doses for 78% of the respondents in the Soso area and more than 99% of respondents in Iwaki were also less than 1 mSv. The dose distribution for all respondents was: 62.0%, <1 mSv; 94.0%, <2 mSv; 99.4%, <3 mSv.

The average and maximum doses (four-month doses, March 11 to July 11, 2011) for each area are also shown in Table 1. The highest maximum dose (25 mSv) for all the respondents was found in the Soso area, where the Fukushima Dai-ichi NPP is located. The average dose for the Soso area was 0.8 mSv, while it was 1.4 mSv for the Kempoku area and 1.0 mSv for the Kenchu area. The average dose for all the respondents was 0.8 mSv.

Figures 3 and 4 (a) show that the gamma ray dose rate was the highest in the Soso area followed by the Kempoku and the Kenchu area. Most of the Soso area was designated as the Evacuation Area or Deliberate Evacuation Area. Many of the Soso residents evacuated from their original location, which is a possible reason for their average dose being lower than the average doses for residents of the Kempoku and Kenchu areas. The average doses for Aizu (0.2 mSv) and Minami-Aizu (0.1 mSv) areas were smaller than those for other areas due to their greater distance from the Fukushima Dai-ichi NPP.

A blown-up map of the area with the highest gamma ray dose rate is shown in Fig. 4(b)^15^. Table 2 lists the dose distribution for each of the six municipalities shown in Fig. 4(b). A more detailed dose distribution is shown in Table S1 in the Supplementary Information. As shown in Table S1, most of the higher doses were reported in Iitate Village and Namie Town. Figure 4(b) indicates that the gamma ray dose rate was generally higher in Namie Town, with similar individual doses seen in Iitate Village (Table S1).

Tables 3 and 4 show dose distributions by age groups and sex, respectively. Some information from mass media accelerated public concerns about exposure to children, especially in the early stage post accident^16^. Doses in children (age groups of 0–9 and 10–19), however, seemed to be similar to those in adults. Children (<20 y) had a dose distribution as follows: 62.6%, <1 mSv; 92.7%, <2 mSv; and 99.6%, <3 mSv. The dose distribution for adults was: 61.7%, <1 mSv; 94.5%, <2 mSv; and 99.2%, <3 mSv. There was little difference in dose distribution between males and females, as seen in Table 4.

## Discussion

The main features of the Basic Survey can be summarized as follows: (1) it is a large scale survey targeting all residents in Fukushima Prefecture including children; (2) the estimated individual dose was mailed to each respondent as a health care service on a personal basis; and (3) a dose estimation method that takes the “personal trail” of moves and activities into account was adopted. Since the Fukushima NPP accident, many dose estimation studies have been performed. Regarding the first two features mentioned above, the Basic Survey is the only one to include both features. Out of 541,653 responses, individual doses have been reported to 491,093 respondents as of June 30, 2014. The responses for infants were written by their parents, but the responses generally covered all age groups, as shown in Table 3.

As for the third feature, most of the studies for estimating the initial dose after the accident used assumptions on moves and a daily time-budget (time spent indoors or outdoors) for residents. The common assumptions were that (1) people stayed outdoors the entire day, and (2) people stayed outdoors 8 hours and 16 hours indoors^12^,^17^. For reference, UNSCEAR used assumptions on indoor occupancy factors of 0.9 for indoor workers, 0.7 for outdoor workers and 0.8 for preschool children^10^. The individual external dose was affected by the gamma ray dose rate at the resident’s original place as well as the time until the start of evacuation. In this respect, the behavior data for each resident were important for individual dose estimation. Since the present study took the behavior data of residents into consideration, accuracy could be higher than in other studies that included such simple assumptions on behaviors of residents.

However, the dose estimation by the present study also includes uncertainties. They mainly come from (1) the fact that behavior data were based on replies to the self-administered questionnaire, and (2) the presence of uncertainty over the gamma ray dose rate maps. Regarding the former, estimation of the personal trail of moves using GPS data was proposed by Hayano and Adachi^18^. Such an approach could be more accurate than the present method, which relies on the resident’s memory of their behavior. For the GPS method, however, the number of available subjects is limited to those who subscribe to the of “Auto-GPS” mobile phone service (about 0.7% of residents in Fukushima Prefecture). Also, due to massive power failures after the accident, it was unlikely that mobile phones were kept charged. When considering that all the residents including all age groups were the target, the present method could be the best choice. Akahane *et al.* mentioned the second source of uncertainty (gamma ray dose rate maps)^11^. They pointed out that: (1) simulation data were used for constructing the maps for the first three days instead of monitoring data; and (2) the gamma ray dose was assumed to be uniform within a 2 km × 2 km mesh.

The four-month doses estimated in the present study were compared with the four-month doses estimated for 18 evacuation patterns of residents from areas within a 20 km radius of the NPP, and the Deliberate Evacuation Area^11^. In their study, two places where the residents started to evacuate were assumed; the representative place (such as the town office), and the place where the dose was highest in the town until the time of evacuation. A comparison between the doses for the first cases (evacuation from the representative places) and the present results was made in the following way:

In the case of Iitate Village, doses for the first representative case (fully evacuated on May 29) and the second representative case (fully evacuated on June 21) were calculated to be 5.5 mSv and 6.2 mSv, respectively. A typical dose estimated by the Basic Survey was generally lower than these figures, as shown in Tables 2 and S1. According to the survey of evacuees, almost 50% of the residents of Iitate Village started to evacuate around March 20^19^. The percentage was roughly 70% on May 29 and more than 80% on June 21. On average, the actual evacuation seemed to start earlier than the assumption made in the above calculations. This could be a reason for the doses by the Basic Survey being generally lower than the representative case doses.

On the other hand, for Namie Town, the dose for the first representative case (evacuated on March 16 from the first designated evacuation center) was calculated to be 2.0 mSv. However, the first designated evacuation center was located in a relatively high dose area, so another scenario (staying there until 10 a.m. on March 23) was calculated, which gave a dose of around 4.8 mSv^11^. According to the survey of evacuees from Namie Town, most of the residents (more than 90%) started to evacuate homes by March 15, but it was not clear from the report when the residents fully evacuated from the Deliberate Evacuation Area^19^. According to the present results, a typical dose seemed to be slightly lower than the dose in the first scenario. However, higher doses could be seen in Namie Town residents, as shown in Table S1. This may have been due to the delayed evacuation by some of the residents. A more detailed analysis of the relationship between the records of moves and activities and individual doses will be conducted later.

The first-year dose after the accident was estimated by several other studies, as described later. For study comparison, the first-year dose was estimated based on the results of the present study in the following way:

Regarding the estimation of external dose four months after the accident, available data can be seen in the form of results for personal dosimeter measurement (the usual integral time of measurement was 2–3 months), which many municipals in Fukushima Prefecture started to conduct several months after the accident. Assuming that short-lived radionuclides had almost decayed four months after the accident (changes in external dose rate reach a plateau), the eight-month external dose following the first four months could be calculated by the results of personal dosimeter measurements^4^. The highest district average dose based on personal dosimeter measurement in 2011 was found in Date City and its three-month average dose was 0.71 mSv^5^. This corresponds to around 1.9 mSv for an eight-month dose. Since Table 1 indicates that the highest four-month dose on area-average basis was 1.4 mSv for the Kempoku area (including Date City), the highest first-year external dose could be around 3 mSv on a district average basis. As for internal dose, whole-body counting results in Fukushima Prefecture showed that the committed effective dose due to cesium (^134^Cs and ^137^Cs) was less than 1 mSv in most cases^20^. Even including internal exposure, the first-year area-average dose could be at most 4 mSv for non-evacuated areas.

On the other hand, residents in the evacuation areas have moved to non-evacuated areas. In the present study, higher doses (>15 mSv) can be seen mainly in persons with delayed moves from evacuation areas after the accident. The first-year dose for these evacuees could be the sum of the four-month external dose and an eight-month external dose of up to 3 mSv received in non-evacuated areas. Since the highest four-month dose was 25 mSv, a first-year dose of 30 mSv at its highest, even internal exposure due to cesium (the maximum for the whole of Fukushima Prefecture was at most 2–3 mSv^20^) was considered.

As mentioned before, the WHO stated that the preliminary estimated effective doses for the first year ranged from 12 to 25 mSv (including internal dose) in Namie Town and Iitate Village^9^. According to the results for whole-body counting, effective doses due to ^134^Cs and ^137^Cs were less than 1 mSv in most cases^3^,^20^. The present results (Table 2 and S1) showed that the majority of doses were <1 mSv and 2–3 mSv for Namie Town and Iitate village, respectively. Also, the gamma ray dose rate decreased with time, as shown in Fig. 2. Thus, considering the present results, the lower end of the range (12 mSv) seemed to be higher than the actual values. Brumfiel estimated that residents of Namie Town and Iitate Village received effective doses of 10–50 mSv for one year after the accident^21^. The residents of the rest of Fukushima Prefecture received effective doses of 1–10 mSv over the same period. Similarly to the estimation by the WHO, the lower end of the range (10 mSv) for one-year dose seemed to be larger than expected from the present results. Also, the higher end of the range (50 mSv) is rather unlikely to be reached, considering the maximum dose estimated in the present study.

Hosoda *et al.* estimated one-year cumulative external doses for evacuees to Fukushima City, Koriyama City and Nihonmatsu City from the residential area where the maximum dose was measured in Namie Town^17^. The doses were 57–68 mSv, 57–59 mSv and 59–64 mSv, respectively, including consideration of a shielding factor of 0.4 for wooden houses and a daily time-budget (outdoors: 8 h and indoors: 16 h)^12^. The estimation by Hosoda *et al.* included the assumption that evacuees were living at the maximum dose rate point for two months after the accident. This assumption may have led to their values being higher than the maximum dose estimated by the first-year dose estimated in the present study (around 30 mSv).

When comparing the district or settlement average effective doses that the UNSCEAR report estimated (1.1–9.3 mSv for adult evacuees and 1.0–4.3 mSv for adult non-evacuees for the first year), higher doses were found in the present study. Since the Basic Survey is based on a questionnaire to individuals, doses for some who did not follow typical evacuation patterns could be revealed. This is one of the features of the Basic Survey, as mentioned at the beginning of this section before. The health implications from the Basic survey were discussed in the following way:

The WHO report suggests that lifetime (or long-term) dose for non-evacuated areas could be estimated by doubling the first-year dose in the case of the Fukushima accident (remediation is considered)^9^. The WHO uses it for calculating lifetime risk. On the other hand, according to the UNSCEAR 2013 report, lifetime doses were estimated to be up to a three-fold greater than the doses received in the first year. However, remediation effects are not considered in this estimation. Since remediation countermeasures have been made in Fukushima Prefecture, it would be reasonable to use the ratio of two suggested by the WHO report. Thus, the highest lifetime district-average dose for non-evacuated areas could be, at most, 8 mSv.

On the other hand, residents of evacuation zones have moved to non-evacuated areas. In the present study, higher doses (>15 mSv) can be seen mainly for persons with delayed moves from the evacuation areas after the accident. If the evacuees continue to stay in non-evacuated areas, their doses in subsequent years beyond the first-year could be around 4 mSv at most, which was equal to the first-year dose for non-evacuated areas, based on the ratio estimated by the WHO report. Thus, the lifetime effective dose could be at most 35 mSv even for adults with the highest four-month dose of 25 mSv (first-year dose was 30 mSv, as mentioned before). For children, the highest four-month dose was within a range of 10–11 mSv for each group of ages 0–9 and 10–19. Thus, the lifetime effective dose for children will be lower than that for the adult.

The UNSCEAR 2013 report states as follows: “while there is little or no direct evidence for an increase in the incidence of cancer as a whole in adult populations from homogeneous whole-body exposures with effective doses of 100 mSv or less, risks can be inferred at such levels (e.g. using a linear dose–response model). However the values of inferred risks are so small that in general no discernible radiation-related increase of overall cancer incidence would be expected among exposed members of the general public”.

Even for persons who were found to have received the highest dose in the Basic Survey could have lifetime doses of far below than 100 mSv. Thus, no discernible increased incidence of radiation-related health effects is expected, as the UNSCEAR 2013 report suggested. However, the UNSCEAR 2013 report also states that separate consideration of certain malignancies has been made for particular groups. For example, for 1-year-old infants who were evacuated after the accident, the UNSCEAR Committee estimated settlement-average absorbed doses to the thyroid to be up to about 80 mGy. An increased risk of thyroid cancer can be inferred for infants and children.

Although the present study deals with external dose only, internal dose to the thyroid is another important topic. The external effective dose can be converted to thyroid equivalent dose due to external radiation using coefficients given by ICRP^22^. The results in the present study will therefore be useful for estimating individual thyroid doses, if the internal dose to the thyroid is estimated on a personal basis. New information on internal dose to the thyroid became available after the UNSCEAR Committee completed its dose estimation^23^,^24^. Combining these results may lead to the refinement of thyroid dose estimation.

The Basic Survey is based on a self-administered questionnaire and participation in the survey is not compulsory. As mentioned before, as of June 30, 2014, the overall response rate is 26.4%. There is room for discussion about the representativeness of responses collected so far, although the ratio of higher doses was very small (0.2%, >5 mSv) according to the present results. The UNSCEAR 2013 report also states that better characterizing distributions of doses to the public is one of the key priorities for scientific research. Possible approaches are collecting more available data on individual behaviors and probabilistic approaches including random sampling of residents and collecting responses from them.

## Additional Information

**How to cite this article**: Ishikawa, T. *et al.* The Fukushima Health Management Survey: estimation of external doses to residents in Fukushima Prefecture. *Sci. Rep.* **5**, 12712; doi: 10.1038/srep12712 (2015).

## Supplementary Material

Supplementary Information

## Figures and Tables

**Table 1:** Distribution of estimated external doses by area.

Effective dose (mSv)	Number of respondents (excluding radiation workers) by areas							Total	
	Kempoku	Kenchu	Kennan	Aizu	Minami-Aizu	Soso	Iwaki	Number	Ratio (%)
<1	23,669	53,547	21,892	37,114	3,775	54,509	66,634	261,140	62.0
1–2	77,265	41,613	2,826	254	29	12,266	595	134,848	32.0
2–3	13,811	7,115	12	16	0	1,621	25	22,600	5.4
3–4	433	369	0	1	0	576	3	1,382	0.3
4–5	39	5	0	0	0	449	1	494	0.1
>5	29	2	0	0	0	898	1	930	0.2
Total	115,246	102,651	24,730	37,385	3,804	70,319	67,259	421,394	100.0
Maximum dose (mSv)	11	5.9	2.6	3.6	1.9	25	5.9	–	–
Average dose (mSv)	1.4	1.0	0.6	0.2	0.1	0.8	0.3	–	–

**Table 2:** Distribution of estimated external doses for each of six municipalities shown in Fig. 4(b).

Effective dose (mSv)	Number of respondents (excluding radiation workers) by areas					
	Minami-Soma	Iitate	Kawamata	Namie	Katsurao	Futaba
<1	18,647	196	613	5,855	493	2,644
1-2	6,046	323	2,634	1,972	158	463
2-3	493	360	174	354	24	72
3-4	95	338	52	64	4	18
4-5	35	357	17	37	0	6
>5	15	731	9	94	1	19
Total	25,331	2,305	3,499	8,376	680	3,222

**Table 3:** Distribution of estimated external doses by age groups.

Effective dose (mSv)	Number of respondents (excluding radiation workers) by age groups (years)									Total
	0–9	10–19	20–29	30–39	40–49	50–59	60–69	70–79	80–	
<1	40,902	36,957	19,572	31,397	26,403	30,692	34,125	24,373	16,719	261,140
1–2	19,529	17,869	9,374	16,982	15,813	17,861	18,719	11,854	6,847	134,848
2–3	5,289	3,297	1,032	2,151	2,087	2,789	3,231	1,904	820	22,600
3–4	216	137	76	147	143	224	216	156	67	1,382
4–5	19	45	36	40	76	90	77	72	39	494
>5	23	31	51	77	99	220	196	157	76	930
Total	65,978	58,336	30,141	50,794	44,621	51,876	56,564	38,516	24,568	421,394

**Table 4:** Distribution of estimated external doses by sex.

Effective dose (mSv)	Male		Female		Total	
	Number^a^	Ratio	Number^a^	Ratio	Number^a^	Ratio
<1	116,202	60.3	144,938	63.4	261,140	62.0
1-2	62,493	32.4	72,355	31.6	134,848	32.0
2-3	12,315	6.4	10,285	4.5	22,600	5.4
3-4	889	0.5	493	0.2	1,382	0.3
4-5	276	0.1	218	0.1	494	0.1
>5	511	0.3	419	0.2	930	0.2
Total	192,686	100.0	228,708	100.0	421,394	100.0

^a^“Number” indicates “Number of respondents (excluding radiation workers)”.
