# Supplementary material for: The Fukushima Health Management Survey: estimation of external doses to residents in Fukushima Prefecture
Source: Sci Rep. 2015 Aug 4;5:12712. doi: 10.1038/srep12712 (PMC4523853; doi:10.1038/srep12712)
Supplement: Supplementary Information [file srep12712-s1.pdf]

Supplementary information

Title

The Fukushima Health Management Survey: estimation of external doses to residents in Fukushima Prefecture

Author names

Tetsuo Ishikawa[1], Seiji Yasumura[1], Kotaro Ozasa[2], Gen Kobashi[3], Hiroshi Yasuda[3], Makoto Miyazaki[1], Keiichi Akahane[3], Shunsuke Yonai[3], Akira Ohtsuru[1], Akira Sakai[1], Ritsu Sakata[2], Kenji Kamiya[1,4] & Masafumi Abe[1]

Affiliations

[1]Radiation Medical Science Center for the Fukushima Health Management Survey, Fukushima Medical University

[2]Department of Epidemiology, Radiation Effects Research Foundation

[3]National Institute of Radiological Sciences

[4]Research Institute for Radiation Biology and Medicine, Hiroshima University

Corresponding author: Tetsuo Ishikawa (isikawat@fmu.ac.jp)

Table S1 Detailed distribution of estimated external doses for each of six municipalities shown in Fig. 4(b)

Effective dose (mSv)	Number of respondents (excluding radiation workers) by areas					
	Minami-Soma	Iitate	Kawamata	Namie	Katsurao	Futaba
<1	18,647	196	613	5,855	493	2,644
1 - 2	6,046	323	2,634	1,972	158	463
2 - 3	493	360	174	354	24	72
3 - 4	95	338	52	64	4	18
4 - 5	35	357	17	37	0	6
5 - 6	3	321	5	17	1	4
6 - 7	6	184	3	15	0	3
7 - 8	4	84	0	12	0	6
8 - 9	1	57	0	9	0	2
9 - 10	0	29	0	5	0	1
10 - 11	0	21	0	10	0	0
11 - 12	1	17	1	8	0	2
12 - 13	0	8	0	5	0	0
13 - 14	0	4	0	4	0	0
14 - 15	0	3	0	3	0	0
> 15	0	3	0	6	0	1
Total	25,331	2,305	3,499	8,376	680	3,222

From the next page, a complete form of the questionnaire is shown as Sheet S1.

The Fukushima Health Management Survey

Basic Survey Medical Interview Sheet (Response Sheet)

00X0X0X

This medical interview sheet is to ask you to record the time and whereabouts during the period from the March 11 earthquake until July 11 (“Record of Movements”) in order to provide a basis for estimating levels of radiation exposure and to assist in future health control.

In particular, since there is no way to evaluate levels of external radiation exposure (other than by estimations based on your “Record of Movement”), we request that you enter your information as accurately as possible, while referring to resources such as the “Events Calendar” contained in the envelope.

The personal information contained in this medical interview sheet will be used for the purpose of health control in Fukushima Prefecture and information will also be provided to the municipality in which you live upon request. However this information will not be disclosed in any way that will identify an individual.

With the exceptions detailed below, this medical questionnaire must be completed only by the named respondent. However, in the case of minors, it should be filled out with a guardian’s consent, and we ask that the guardian’s name be written in the signature block provided for the guardian on the next page. For children under elementary school age, this form should be filled out by a guardian. Furthermore, for persons who are unable to complete the questionnaire by themselves (e.g., the elderly), it must be completed by a family member or a person living with the respondent.

After completing the questionnaire, please enclose it in the provided return envelope and return by post. It is acceptable to return multiple forms, such as those of family members, together in a single envelope.

Fukushima Prefecture

Fukushima Medical University

Please fill out the required information in the underlined spaces and select the applicable boxes with a tick (✔).

Today's Date:<br>2011 Month______Day______	Respondent:<br>1☐ Self 2☐ Proxy (Relationship__________)
Name (Family name, First Name):<br>Katakana (if applicable):	Gender:<br>1☐Male<br>2☐Female
Date of Birth : Year__________Month__________Date_______	

Guardian Signature Block (If the respondent is a minor, when consenting to complete this survey, please provide the name and signature of the guardian)

(Guardian's printed name) (Guardian's signature)

Place of Birth (Nationality):

___________________ Prefecture ____________________ City/Ward/County

_________________Ward/Town/Village ________________

Registered address shown in the Alien Registration card as of March 11, 2011:

〒_______ — _______

1☐ Same as on the cover (go to the next question)

2☐ Same as place of birth (go to the next question)

_________________ Prefecture ____________ City/Ward/County __________Ward/Town/Village

Name of Building / Room Number: ____

Current address:

〒_______ — _______

1☐ Same as place of birth (go to the next question)

2☐ Same as place of birth (go to the next question)

3☐ Same as on March 11 (go to the next question)

________________ Prefecture ____________ City/Ward/County___________Ward/Town/Village

Name of Building / Room Number:____

Intended new address (Please fill this out if you know the address to which you plan to move in the future)

〒_______ — _______

_________________ Prefecture ____________ City/Ward/County ___________Ward/Town/Village

Name of Building / Room Number:____

Intended date for moving: Year________Month________Date________

Contact Information

*This is necessary in case we need to contact the respondent directly, such as to confirm incomplete information.

Phone Number: (______) ______-______ Mobile Phone Number: (______) ______-______

Are you currently living with others?

1☐ I live alone 2☐ I live with my family (including living only with spouse)

3☐ I am living in a facility 4☐ Other (_________________________________)

1. Please mark the appropriate box with a tick (✔) with respect to the building type of your home and the place of employment/education as of March 11.

In the space provided for place of employment/education, please provide details of the building type for other paces (apart from your home) where you spend a lot of time as part of your daily routine, such as for employment or schooling. Please provide details of the address up to the city and neighborhood (chōme) or village level, as appropriate.

For fields labeled “Other” please provide specific details regarding building construction.

If you work outdoors, please select the “Other” box with a tick (✔).

<table>
<tr><td>Home</td><td>1☐ Detached house (reinforced/steel-reinforced concrete construction)
2☐ Detached house (other construction, e.g., wood, steel, light-gauge steel)
3☐ Multiple dwelling/apartment (reinforced/steel-reinforced concrete construction)
(floor ___ of a ___ floor building)
4☐ Multiple dwelling/apartment (other construction, e.g., wood, steel, light-gauge steel)
(floor ___ of a ___ floor building)
5☐ Other (__________) (floor ___ of a ___ floor building)

*For the purpose of this survey, please answer “concrete” for all buildings of the following construction types: reinforced concrete; steel; and concrete block.</td></tr>
<tr><td rowspan="3">Place of employment/ education</td><td>1☐Place of Employment 2☐School 3☐Other (__________________)</td></tr>
<tr><td>Address of place of employment or school (to the best of your knowledge)

____________________ Prefecture ____________________City/Ward/County
____________________Ward/Town/Village____________________</td></tr>
<tr><td>1☐ Reinforced/steel-reinforced concrete construction
(floor ___ of a ___ floor building)

2☐ Other construction (e.g., wood, steel, light-gauge steel)
(floor ___ of a ___ floor building)

3☐ Outdoors (agriculture, etc.)

If you have filled out details in this section, please use labels such as “Employment” and “Education” for the movement survey in the following Section 2.</td></tr>
</table>

2. We would like to know the locations and the relevant lengths of time with respect to places you visited in March this year. Please provide information about your movements between March 11 and March 25, following the example below.

Example

- Please use arrows to indicate durations of time. If you spent time away from your place of residence, please provide details of the place name up to the city and neighborhood (*chōme*) or village, as appropriate.
- For schools, public institutions, etc. it is sufficient to provide the name of the facility only.
- Please provide information for each category: Indoors, Moving, and Outdoors. For time spent indoors, please indicate whether this was in a wood or concrete building by marking with "(W)" or "(C)", respectively.
- Please note that it is not necessary to state whether it was a wood or concrete building with respect to your home or place of work.
- Please indicate the time spent outdoors next to the arrow and provide details about the location in the right column.
- For time spent outside Fukushima Prefecture, please indicate the total time, including travel time or time spent outdoors, under the section marked "Whereabouts: Indoors".

	Whereabouts	Time<br>0 3 6 9 12 15 18 21 24	Place name / Facility
Example	Indoors	① ④ ④	① Home<br>② Your own field/garden<br>③ Car<br>④Evacuation centre<br>(△ Jr High School,<br>○○ -shi) (C)<br>⑤△-aza, ○○-machi, ○○-shi
	Moving	③	
	Outdoors	②(1 hour) ⑤(2.5 hours)	

Actual movements

	Whereabouts	Time<br>0 3 6 9 12 15 18 21 24	Place / Facility
March 11 (Fri)	Indoors		
	Moving		
	Outdoors		
March 12 (Sat)	Indoors		
	Moving		
	Outdoors		
March 13 (Sun)	Indoors		
	Moving		
	Outdoors		
March 14 (Mon)	Indoors		
	Moving		
	Outdoors		
March 15 (Tue)	Indoors		
	Moving		
	Outdoors		

Actual movements (continued)

	Whereabouts	Time 0 3 6 9 12 15 18 21 24	Place / Facility
March 16 (Wed)	Indoors		
	Moving		
	Outdoors		
March 17 (Thu)	Indoors		
	Moving		
	Outdoors		
March 18 (Fri)	Indoors		
	Moving		
	Outdoors		
March 19 (Sat)	Indoors		
	Moving		
	Outdoors		
March 20 (Sun)	Indoors		
	Moving		
	Outdoors		
March 21 (Mon)	Indoors		
	Moving		
	Outdoors		
March 22 (Tue)	Indoors		
	Moving		
	Outdoors		
March 23 (Wed)	Indoors		
	Moving		
	Outdoors		
March 24 (Thu)	Indoors		
	Moving		
	Outdoors		
March 25 (Fri)	Indoors		
	Moving		
	Outdoors		

3. We would like to ask you about places you visited and places of regular outings for the period from March 26 to July 11, 2011. For example, if you commuted to work from Monday to Friday and you had different regular outings on Saturdays, please fill in the former as "Outing (1)" and the latter as "Outing (2)" etc.

Please fill in the address details up to the house/block number (*banchi*). If you are unsure of the address of a place which you visited while on an outing, it is acceptable to provide just the name of the facility. Also, for visits outside Fukushima Prefecture, it is acceptable to provide the address up to the name of the municipality.

Period	**Places where you spent time**
March 26<br>⇩<br>*(month)* *(day)*	Area of residence<br>_____ Prefecture______City/Ward/Parish________Ward/Town/Village<br>Time spent outside for shopping, doing chores, etc in the area surrounding your place of residence: [________] hours per day<br><br>Place of regular outing ① (place of employment or education):<br>Facility name:______________________________<br>Location:<br>_____ Prefecture______City/Ward/Parish________Ward/Town/Village<br>Time spent indoors at the place for outing: [________] hours per day<br>Time spent outdoors at the place for outing: [________] hours per day<br>Please circle the relevant day(s): Mon Tue Wed Thu Fri Sat Sun<br><br>Place of regular outing ②: Facility name______________________<br>Location:<br>_____ Prefecture______City/Ward/Parish________Ward/Town/Village<br>Time spent indoors at place of outing: [________] hours per day<br>Time spent outdoors at place of outing: [________] hours per day<br>Please circle the relevant day(s): Mon Tue Wed Thu Fri Sat Sun
(month) *(day)*<br>⇩<br>*(month)* *(day)*	Area of residence<br>_____ Prefecture______City/Ward/Parish________Ward/Town/Village<br>Time spent outside for shopping, doing chores, etc in the area surrounding your place of residence: [________] hours per day<br><br>Place of regular outing ① (place of employment or education):<br>Facility name:______________________________<br>Location:<br>_____ Prefecture______City/Ward/Parish________Ward/Town/Village<br>Time spent indoors at the place for outing: [________] hours per day<br>Time spent outdoors at the place for outing: [________] hours per day<br>Please circle the relevant day(s): Mon Tue Wed Thu Fri Sat Sun<br><br>Place of regular outing ②: Facility name______________________<br>Location:<br>_____ Prefecture______City/Ward/Parish________Ward/Town/Village<br>Time spent indoors at place of outing: [________] hours per day<br>Time spent outdoors at place of outing: [________] hours per day<br>Please circle the relevant day(s): Mon Tue Wed Thu Fri Sat Sun

Period	**Places where you spent time**
(month) *(day)*<br>⇩<br>*(month)* *(day)*	Area of residence<br>_____ Prefecture_______City/Ward/Parish_________Ward/Town/Village<br>Time spent outside for shopping, doing chores, etc in the area surrounding your place of residence: [_________] hours per day<br><br>Place of regular outing ① (place of employment or education):<br>Facility name:____<br>Location:<br>_____ Prefecture_______City/Ward/Parish_________Ward/Town/Village<br>Time spent indoors at the place for outing: [_________] hours per day<br>Time spent outdoors at the place for outing: [_________] hours per day<br>Please circle the relevant day(s): Mon Tue Wed Thu Fri Sat Sun<br><br>Place of regular outing ②: Facility name_________________________________<br>Location:<br>_____ Prefecture_______City/Ward/Parish_________Ward/Town/Village<br>Time spent indoors at place of outing: [_________] hours per day<br>Time spent outdoors at place of outing: [_________] hours per day<br>Please circle the relevant day(s): Mon Tue Wed Thu Fri Sat Sun
(month) *(day)*<br>⇩<br>*(month)* *(day)*	Area of residence<br>_____ Prefecture_______City/Ward/Parish_________Ward/Town/Village<br>Time spent outside for shopping, doing chores, etc in the area surrounding your place of residence: [_________] hours per day<br><br>Place of regular outing ① (place of employment or education):<br>Facility name:____<br>Location:<br>_____ Prefecture_______City/Ward/Parish_________Ward/Town/Village<br>Time spent indoors at the place for outing: [_________] hours per day<br>Time spent outdoors at the place for outing: [_________] hours per day<br>Please circle the relevant day(s): Mon Tue Wed Thu Fri Sat Sun<br><br>Place of regular outing ②: Facility name_________________________________<br>Location:<br>_____ Prefecture_______City/Ward/Parish_________Ward/Town/Village<br>Time spent indoors at place of outing: [_________] hours per day<br>Time spent outdoors at place of outing: [_________] hours per day<br>Please circle the relevant day(s): Mon Tue Wed Thu Fri Sat Sun

*Space for four (4) periods is provided; however, if this is insufficient, please submit with additional photocopies, etc., attached.

4. Please fill in the dates and activities for days on or after March 26 to July 11 which do not correspond to the answer in (3) above. For time spent outside Fukushima Prefecture, please include all travel time in the column of “indoors”

Example

- Please use arrows to indicate durations of time. If you spent time away from your place of residence, please provide details of the place name up to the city and neighborhood (*chōme*) or village, as appropriate.
- For schools, public institutions, etc. it is sufficient to provide the name of the facility only.
- Please provide information for each category: Indoors, Moving, and Outdoors. For time spent indoors, please indicate whether this was in a wood or concrete building by marking with “(W)” or “(C)”, respectively.
- Please note that it is not necessary to state whether it was a wood or concrete building with respect to your home or place of work.
- Please indicate the time spent outdoors next to the arrow and provide details about the location in the right column.
- For time spent outside Fukushima Prefecture, please indicate the total time, including travel time or time spent outdoors, under the section marked “Whereabouts: Indoors”.

	Whereabouts	Time<br>0 3 6 9 12 15 18 21 24	Place / Facility
May 1	Indoors	① ④ ④	① Home<br>②Your own field/garden<br>③ Car<br>④ Evacuation centre (△ Jr High School, ○○ -shi) (C)<br>⑤△-aza, ○○-machi, ○○-shi
	Moving	③	
	Outdoors	②(1 hour) ⑤(2.5 hours)	
May 5	Indoors	① ③ ①	①Home<br>②Car<br>③○○ Prefecture
	Moving	② ②	
	Outdoors		
May 8<br>May 15<br>May 22	Indoors		Almost the same as on May 1
	Moving		
	Outdoors		

Activities not corresponding to (3) above.

	Whereabouts	Time<br>0 3 6 9 12 15 18 21 24	Place / Facility
( )	Indoors		
	Moving		
	Outdoors		
( )	Indoors		
	Moving		
	Outdoors		
( )	Indoors		
	Moving		
	Outdoors		

( )	Indoors		
	Moving		
	Outdoors		
( )	Indoors		
	Moving		
	Outdoors		
( )	Indoors		
	Moving		
	Outdoors		
( )	Indoors		
	Moving		
	Outdoors		

*Space for seven (7) days is provided; however, if this is insufficient, please submit with additional photocopies, etc., attached.

5. If you entered the restricted area, such as for temporary access to your home, etc, please enter the date and activities performed on that date.

	Whereabouts	Time<br>0 3 6 9 12 15 18 21 24	Place / Facility
( )	Indoors		
	Moving		
	Outdoors		
( )	Indoors		
	Moving		
	Outdoors		
( )	Indoors		
	Moving		
	Outdoors		

6. From March 11 through to March 31, to what extent did you consume products such as crops produced in your own fields, orchards, home garden, etc, or milk from livestock that you raise? Please mark the appropriate box with a tick (✔) and provide details in the square brackets ([ ]) with respect to types of food eaten, whether it was from an open field or greenhouse, and how many times such foodstuffs were consumed.

Type of food	Classification	
Did you eat any home-grown fruit or vegetables?	1□Yes →<br>2□No ↓<br>Go to the next question.	If you answered "yes", fill in the following information:<br>①Type [ ] : 1□ Field 2□ Greenhouse 3□Unknown<br>Approximately what quantity?<br>Serving size: Small: [ ] Medium: [ ] Large: [ ] ***or*** No. of pieces [ ]<br>②Type [ ] : 1□ Field 2□ Greenhouse 3□Unknown<br>Approximately what quantity?<br>Serving size: Small: [ ] Medium: [ ] Large: [ ] ***or*** No. of pieces [ ]<br>③Type [ ] : 1□ Field 2□ Greenhouse 3□Unknown<br>Approximately what quantity?<br>Serving size: Small: [ ] Medium: [ ] Large: [ ] ***or*** No. of pieces [ ]<br>④Type [ ] : 1□ Field 2□ Greenhouse 3□Unknown<br>Approximately what quantity?<br>Serving size: Small: [ ] Medium: [ ] Large: [ ] ***or*** No. of pieces [ ]<br>⑤Type [ ] : 1□ Field 2□ Greenhouse 3□Unknown<br>Approximately what quantity?<br>Serving size: Small: [ ] Medium: [ ] Large: [ ]***or*** No. of pieces [ ]<br>⑥Type [ ] : 1□ Field 2□ Greenhouse 3□Unknown<br>Approximately what quantity?<br>Serving size: Small: [ ] Medium: [ ] Large: [ ]***or*** No. of pieces [ ]<br>*(A guide for serving sizes should be the normal amount that is served on plates of the following diameters, respectively: less than 10 cm (small), 10-20cm (medium) and over 20 cm (large))*
Did you drink milk from any livestock you raise (cows, etc)?	1□Yes →<br>2□No ↓<br>Go to the next question.	Type of livestock: [ ]<br>On the basis of a 200 mL cup, how many cups in total did you drink?<br>[__________]

7. For the period between March 11 and March 31, which of the following was the main source of drinking water that you used for everyday consumption?

Please mark the appropriate box with a tick (✔) and provide details in the square brackets ([ ])

1☐ Tap water	Consumed daily between: March [__] and March [__]
2☐ Water from private waterworks or well	Consumed daily between: March [__] and March [__]
3☐ Mineral water	Consumed daily between: March [__] and March [__]
4☐ Other [______________________________]	

8. For the period between March 11 and March 31, did you take "stabilized iodine tablet"?

1☐ No 2☐ Yes→ Date taken [_____(Month) _____(Day)] 3☐ I don't know.

9. For the period between March 11 and March 31, did you undergo screening for radioactive contamination?

1☐ No

2☐ Yes → _____(Month) _____ (Day) Place at measurement taken [ ]

Were you decontaminated? 1☐ No 2☐ Yes

10. Are you currently or have you previously been a "radiation worker"?

1☐ No 2☐ Yes

3☐ I don't know → Please describe the details of your occupation (__________________)

This is the end of the survey.
Please submit this form with the return envelope.
